# Capture, analyse, visualise: An exemplar of performance analysis in practice in field hockey

**DOI:** 10.1371/journal.pone.0268171

**Published:** 2022-05-05

**Authors:** Felicity Lord, David B. Pyne, Marijke Welvaert, Jocelyn K. Mara

**Affiliations:** 1 University of Canberra Research Institute for Sport and Exercise (UCRISE), University of Canberra, Bruce, Canberra, Australia; 2 Statistical Consulting Unit, The Australian National University, Canberra, Australia; University of Cassino e Lazio Meridionale, ITALY

## Abstract

The goal of performance analysis is to capture the multitude of factors that affect sports strategy, and present them in an informative, interpretable, and accessible format. The aim of this study was to outline a performance analysis process in field hockey that captures, analyses and visualises strategy in layers of detail culminating in the creation of an RStudio Shiny application. Computerised notational analysis systems were developed to capture in-game events and ball tracking data of 74 matches from the Women’s Pro League 2019. Game styles were developed using k-means cluster analysis to reduce detailed in-game events into practical profiles to identify the attack types, game actions and tempo of a team’s strategy. Ball movement profiles were developed to identify the predictability (entropy) and direction (progression rates) of ball movements, and consequent distribution of possession in different attacking zones. The Shiny application, an interactive web-platform, links the information from simple game profiles with detailed game variables to understand each teams’ holistic game plan, how they are different, and how to exploit these differences. The process outlined can be applied to any team invasion sport to understand, develop and communicate successful strategies under different match situations.

## Introduction

Performance analysis is used to provide insight into tactics and strategy. The role of the performance analyst is defined as translating objective data into learning opportunities to help sport coaches understand how and why outcomes occurred to improve future performance [1]. Study of performance analysis was undertaken as long ago as 1936 with the pioneering work of Dr Anna Espenschade who detailed player demands and movements in field hockey [2]. A large proportion of performance analysis research has focused on identifying differences between successful and less successful teams to identify key performance indicators [3]. These key performance indicators then become the benchmark a team needs to achieve to win a game or tournament [3]. However, outcome-orientated performance indicators only report *what* happened. There is more than one way to win a game and these simple performance indicators do not provide insight into *how* a team achieved these outcomes. Here we focus on process-oriented performance analysis in team invasion sports to provide effective insights into team and coaching strategy.

**Fig 1:**
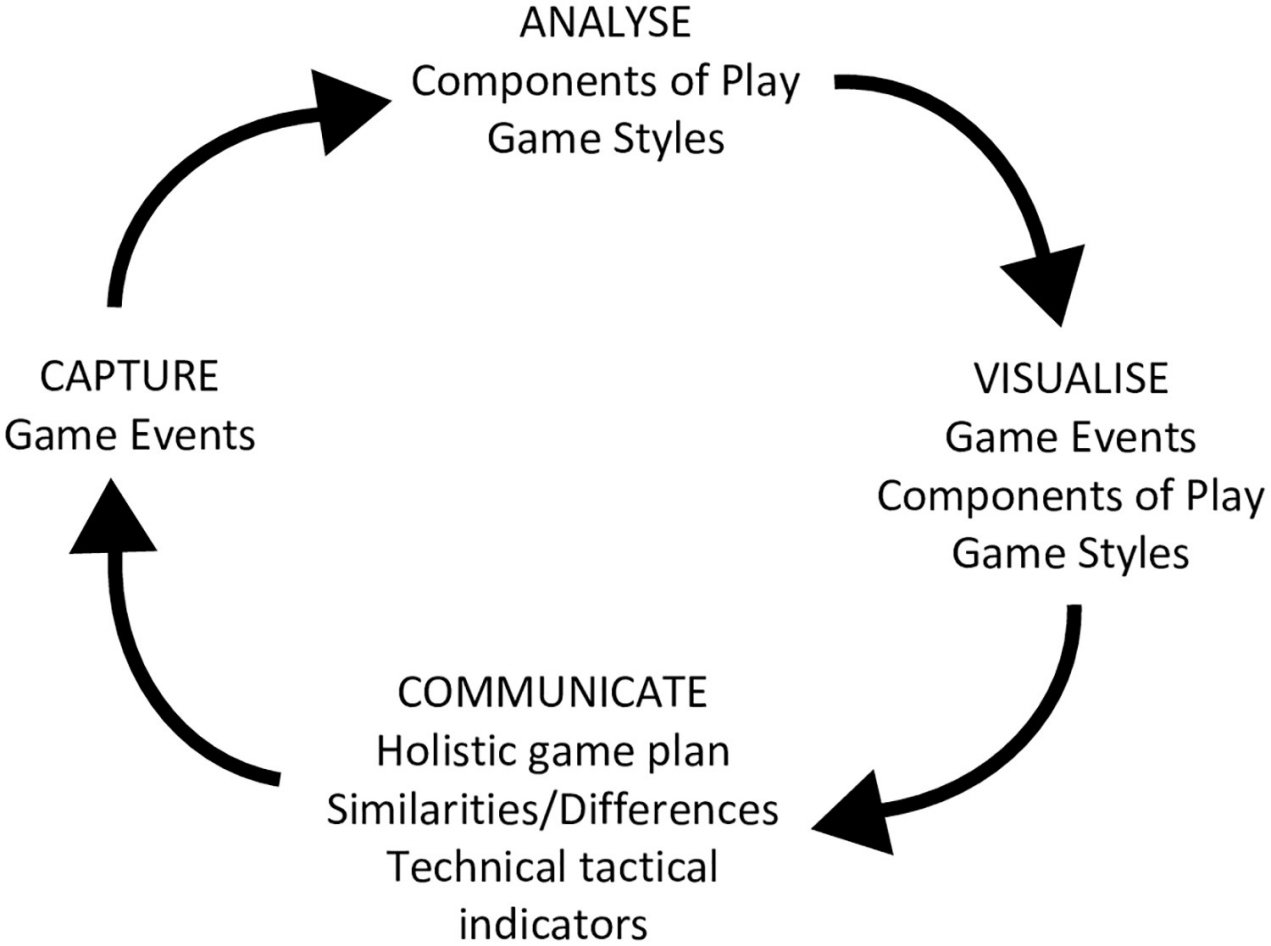
Performance analysis process employed to understand, develop and communicate layers of strategical information.

The main processes in performance analysis are data capture, analysis, visualisation and communication [4, 5]. Appropriate methods must be utilised in each step to ensure the effective communication and translation of practical insights into strategy [1]. To understand team strategy, an analyst and coach must understand what a team is trying to achieve, how they go about achieving it, and the technical-tactical elements that contribute to each step. Therefore, performance analysis should capture, analyse, visualise and communicate multiple layers of information as illustrated in Fig 1. This approach ranges from detailed nuances of teams that help form the building blocks of a strategy, to practical identities that reflect key aspects of performance that can be observed and communicated to players.

Team invasion sports are considered to be dynamic, complex systems [6–9]. A dynamic, complex system reflects an environment with multiple interacting factors that are constantly changing [10]. To provide effective and practical insight into strategy, it is essential to capture in-game events in great detail to replicate the multitude of interacting elements that affect each action and outcome in a game. When analysing data, it is important to generate insights within the context of a moment in a game, and to present the data in meaningful ways that facilitate the development of strategy. This approach means placing greater focus on the issues of *how* and *why* in relation to outcomes. Advancements in technology, such as computerised notational analysis systems and semi-automated tracking systems, have allowed a greater amount of detail to be captured with more emphasis on spatio-temporal variables and opposition interactions [4, 11]. Implementation of data science software and analytic methods has accommodated the increasing number of data points captured per game, and dimension reduction techniques are often employed to reduce large, complex data sets into simple, practical profiles [3, 12]. Use of this software has enhanced the presentation of results using interactive visualisations and dashboards such as analysis of the NCAA 2020 basketball tournament in Google Cloud (https://datastudio.google.com/reporting/1gh6NGVURB3gMcwfh1Ubob474biePAw3U/page/TTCk).

The field of performance analysis has evolved with changing technologies and perspectives, and there is growing interest in each step of the process in research. Key elements include validating data capture tools [13, 14], analysing data to identify game styles [15–17] and describing visualisation tools for live game events [18–20]. However, there is often a disconnect between research and practice with limited guidelines and tools to facilitate a clear and effective approach for conducting performance analysis in the real world. In practice, data capture, analysis and visualisation must occur cyclically [4]. Constraints such as limited resources and time restrictions are imposed, and this process must analyse both the team behaviours in regard to the outcome of a match, and the trends occurring in a tournament or season. Collectively these challenges may limit the effective communication of strategy in team invasion sports.

The aim of this study was to outline a performance analysis process, from data capture to communication, in field hockey for developing and communicating strategy in preparation for upcoming games. The secondary aim was to develop a Shiny application in RStudio (https://shiny.rstudio.com) to illustrate how strategy can be communicated. Data capture was completed with a computerised notational system, and data analysis and visualisation conducted using programming software. Here we provide access to data analysis and visualisation code to demonstrate the technical aspects involved so the process may be reproduced and applied to other sports or tournaments. This process utilises contemporary and emerging analytic techniques that can be completed with limited resources. The key concepts in capturing, analysing, and visualising data can be applied to any team invasion sport. No other study has detailed the process from start to finish so this study showcases the pathway from research to practice in performance analysis and data visualisation.

## Design considerations

### Data capture

**Fig 2:**
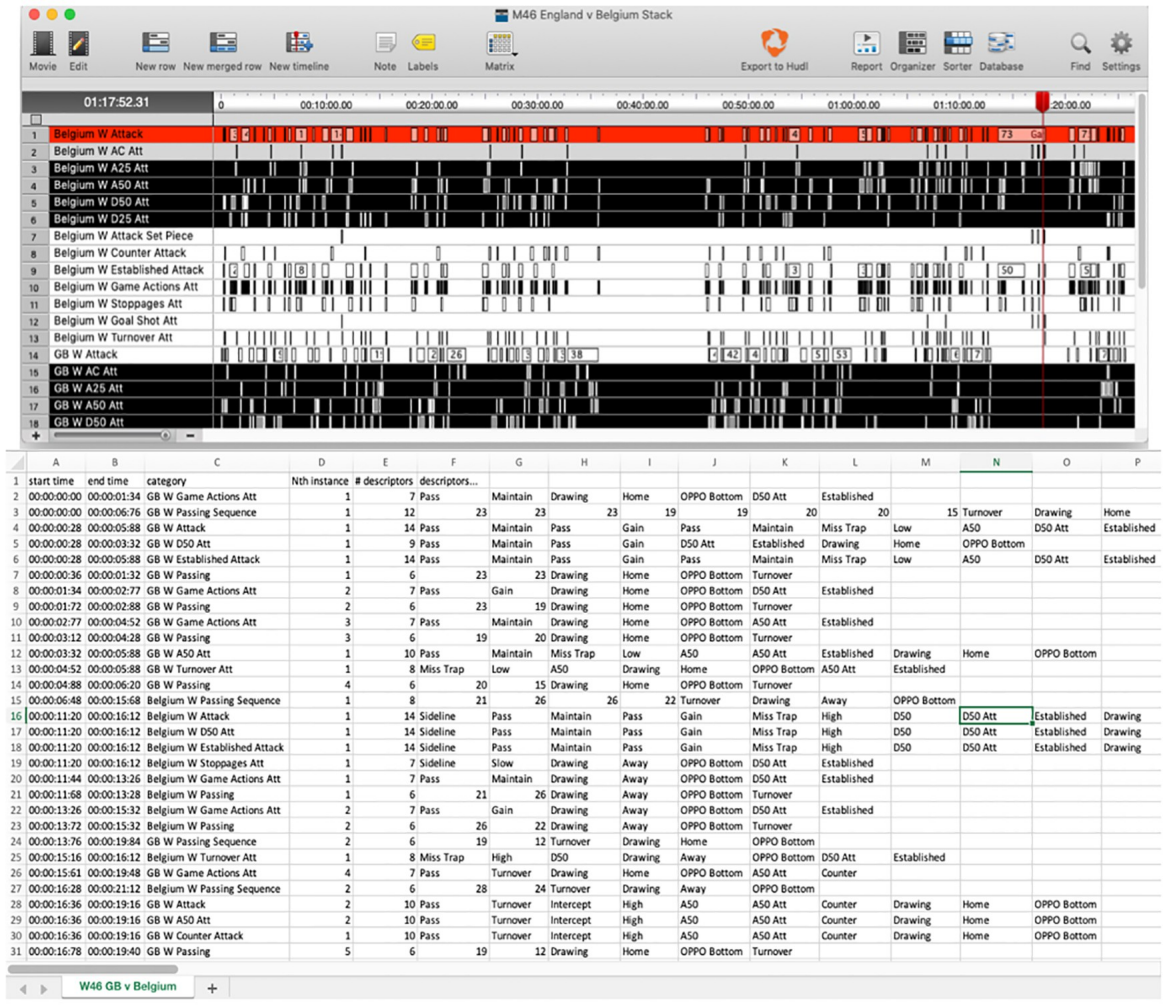
**(A**). **Example of a timeline of coded events in SportsCode**. Each row represents a coded event with each rectangle within a row reflecting an individual instance of that event and includes all descriptive labels. (**B). XML output of example timeline displayed in Microsoft Excel**. Each row represents an instance from a code row (category column) and all included descriptive labels (from descriptors column).

The development of computerised notational analysis systems allows games to be analysed retrospectively, and video to be paused and rewound to ensure a high volume of data is captured accurately. Information is captured using a “code window” that includes code and label buttons. All actions and events deemed important to answer a pre-determined question are recommended to be included as a code button. Labels can be added to code rows as descriptions allowing layers of contextual information to be recorded per event. When a code button is clicked it generates an instance in a separate row in the timeline (Fig 2A). Code rows could range from general to specific, such as a whole possession to an individual game action, so layers of analysis can be easily extracted and analysed. When a label button is clicked the descriptive information is placed in the linked code rows. It is suggested to include labels describing each game event per time, space, opposition and match context, in all code rows.

Before capturing data, the validity of code windows should be assessed to ensure variables captured can be interpreted accurately and notated reliably. Clear and unambiguous definitions must be presented for all descriptive variables to be analysed. When capturing spatial data, natural landmarks on the field, such as the halfway line or goal posts, can be used to divide the field into cells to make capturing the data more reliable. It is suggested that the size of the cells be the smallest dimension(s) that provides meaningful impact when moving between them, and considering the type of skills used to move the ball, and the length of passes achievable. Reliability of categorical variables should be assessed through inter- and intra-observer reliability tests to calculate kappa coefficients, indicating the degree of agreement between observers. A kappa coefficient should be >0.80 to ensure the data capture process is reliable [21].

### Data analysis

The aim of data analysis is to summarise the information captured across a period of time to uncover patterns or trends across games reflecting common strategies used by teams. To provide layers of detailed insight, information can be extracted from the raw data by analysing interactions between variables, and then condensed into practical profiles to reflect the holistic nature of sport [3]. Raw data first needs to be normalised by converting to a ratio or percentage [22]. Each variable can be divided by the total actions or events such as percentage of goal shots per attack type or ball movements per location, to provide context for the value and allow variables to be compared between games. Capturing and analysing in-game events or ball movements in great detail permits targeted analysis for different phases of the game.

To condense highly detailed in-game event variables into a holistic game plan, clustering algorithms can be employed. Clustering algorithms, such as k-means clustering, factor analysis or self-organising maps, group games with similar patterns into clusters which represent different strategies occurring during a game [16, 17, 23, 24]. These patterns are deemed game styles which are defined as the consistent strategy implemented by a team [25]. This process is expediated by employing clustering algorithms using programming software, removing the need for the analyst to make these calculations manually [12]. Data may be divided into pre-determined categories to identify types of strategy within different components of the game such as the “moment of play framework” [25, 26]. Alternatively, a clustering algorithm can determine the categories of performance [16, 17, 24]. Commonly used categories of performance include game actions, attack types and tempo as they reflect the major ways in which a strategy can be manipulated.

It is recommended that variables included in the clustering model provide holistic insight into strategy. Variables that do not provide practically important differences between clusters should be omitted. This approach seeks to avoid over fitting the model, whereby the prediction accuracy of future data sets is reduced [27]. A balance needs to be established between including highly detailed variables, and simplifying or generalising variables, so differences between game style types can be reported. Although individual nuances can be identified by studying a game event in great detail, they might not contribute to overall game style strategy. Similarly, condensing spatial data on ball movements into practical profiles requires areas of the field to be grouped by different phases of attack. This action allows cells on the field with similar purpose and outcomes to be clustered so strategies can be observed in different parts of the game that may be overlooked when viewed in great detail. The attacking zones should stem from deep in defence when a team is under high pressure, to building an attack and developing goal scoring opportunities. The shape and size of the zones must encompass the structure of a team and the types of ball movement occurring in each.

### Data visualisation and communication

Visualisations should allow the viewer to identify trends and patterns at a glance without the viewer having to process large amounts of information [12, 28]. They are the medium by which information on strategies will be communicated. Ineffective visualisations can lead to misinterpretation of data or confusion which limits the work completed to capture and analyse the data in practical ways. For visualisations to be impactful, it is suggested to present data in relation to a standard or reference value so that practical differences can be identified.

Visualisations can be manipulated by the geometry or type of figure used and the aesthetics of the elements in the figure [29]. Perceived aesthetics have been shown to be correlated with user engagement, where users were more likely to spend longer time studying a figure when the design of the visualisation was more pleasing to the eye [30]. Scatter plots can be used to show the relationship between two continuous variables, lines plots may be used to show continuous variables over time, and boxplots, histograms or raincloud plots can be used to present the distribution of continuous variables [31]. Comparatively, the distribution of discrete or categorical variables can be presented in bar plots, and the relationship between two categorical variables in a tile plot.

Aesthetics can be used to add additional dimensions or variables to a figure and include colour, size, and shape [32]. Colour can be used to highlight differences between variables. It is recommended to use a qualitative colour palette to differentiate categorical variables, with a distinct colour employed to separate each level of the variable. A sequential palette is suggested to be used for ordered numeric data, with colour increasing from light to dark to represent low to high numbers or values of greater importance. A divergent palette is advised for numeric values that include both positive and negative values and a central value. A divergent palette essentially contains two sequential palettes that merge in the middle to represent the central value, with lighter colours seen towards the middle and darker representing values further from the midpoint. A colour-blind friendly colour palette is recommended to enhance accessibility for all users. Size and shape can also be manipulated to emphasise different values with shapes identifying different categories and increasing shape size reflecting larger values or importance.

The medium in which the visualisations are presented also influences the insights being communicated. There is a trend away from static visualisations to dynamic web-based platforms given the ability for user interaction [33]. The ability to interact, explore and filter a dataset through the visualisation can improve user engagement [34]. A greater depth of information can be presented in an interactive visualisation as layers of information can be displayed through manipulating the figures inputs. These figures are better able reflect the spatio-temporal and opposition influences on decisions and outcomes in team invasion sport. Accessibility to open-source programming environments such as RStudio has made it easier to produce effective interactive visualisations and share them online for improving communication and translating strategy in practice [12]. These programs also allow the presentation of multiple related figures as a dashboard or application so the bigger picture can be understood by linking the key findings from each visual. When presenting a range of visualisations that revolve around the same theme, it is important to provide consistency between figures so a coach can focus on interpreting the results rather than trying to process the design of the figure. For example, providing a consistent colour palette for variables or unique levels of variables used multiple times (i.e., red indicates losing a match and green indicates winning), and having legends placed in the same location.

## Methods

### Data capture

Video footage from 74 matches from the 2019 International Hockey Federation (FIH) Women’s Pro League were analysed retrospectively using SportsCode (Version 11, Hudl, https://www.hudl.com), a computerised notational analysis software. Each game was reviewed twice to code for in-game events and ball movements separately. Kappa coefficients for intra- and inter-observer reliability tests were >0.88 and >0.86 for in-game event, and >0.91 and >0.87 for ball movement code windows respectively, demonstrating that the data capture tools were reliable [35].

**Fig 3:**
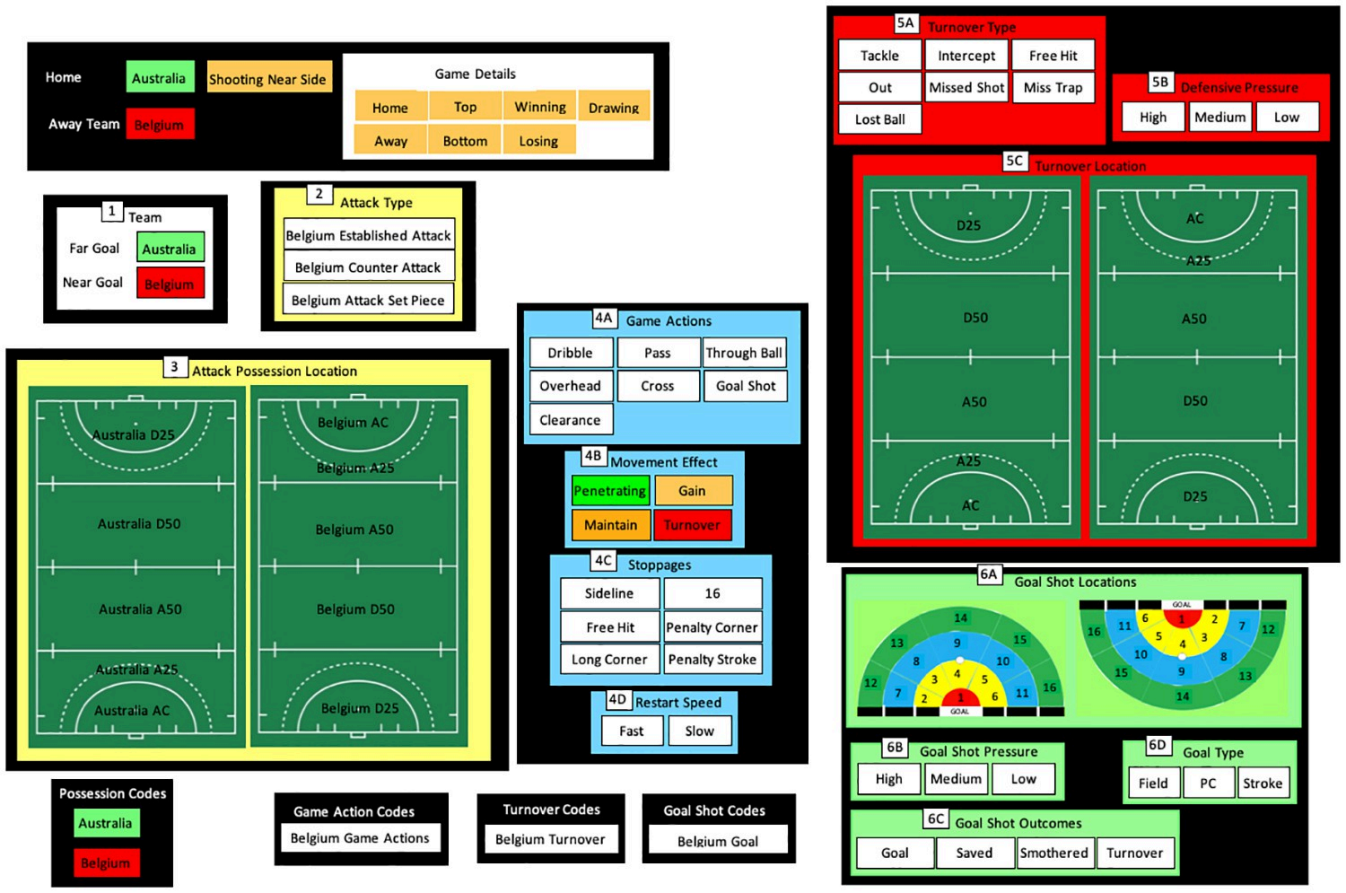
Notational analysis system used to capture game variables in SportsCode to create game styles. The numbered boxes illustrate the steps to follow to code a sequence of field hockey play by selecting the team in possession, attack type, field location, and then options for in-game events available in boxes 4–6. The code window provides an example of a game between Australia (home team) and Belgium (away team).

The code window used to analyse in-game events is displayed in Fig 3. Refer to Lord et al., [23] for a detailed explanation of data capture procedures and variables. Code rows (and their associated labels) included all user-defined major game events; game actions (types and movement effects), stoppages (types and restart speeds), turnovers (types, pressures, and locations) and goal shots (outcomes, pressures, and locations). These code rows also included attack types, field locations, match status, match location and quality of the opposition as labels. All game events and actions were labelled in a team possession code row and divided into attack type and field locations code rows. This process allowed a possession, attack type, field location, or specific game event to be easily and efficiently analysed in great detail using the descriptors of the code row in question.

**Fig 4:**
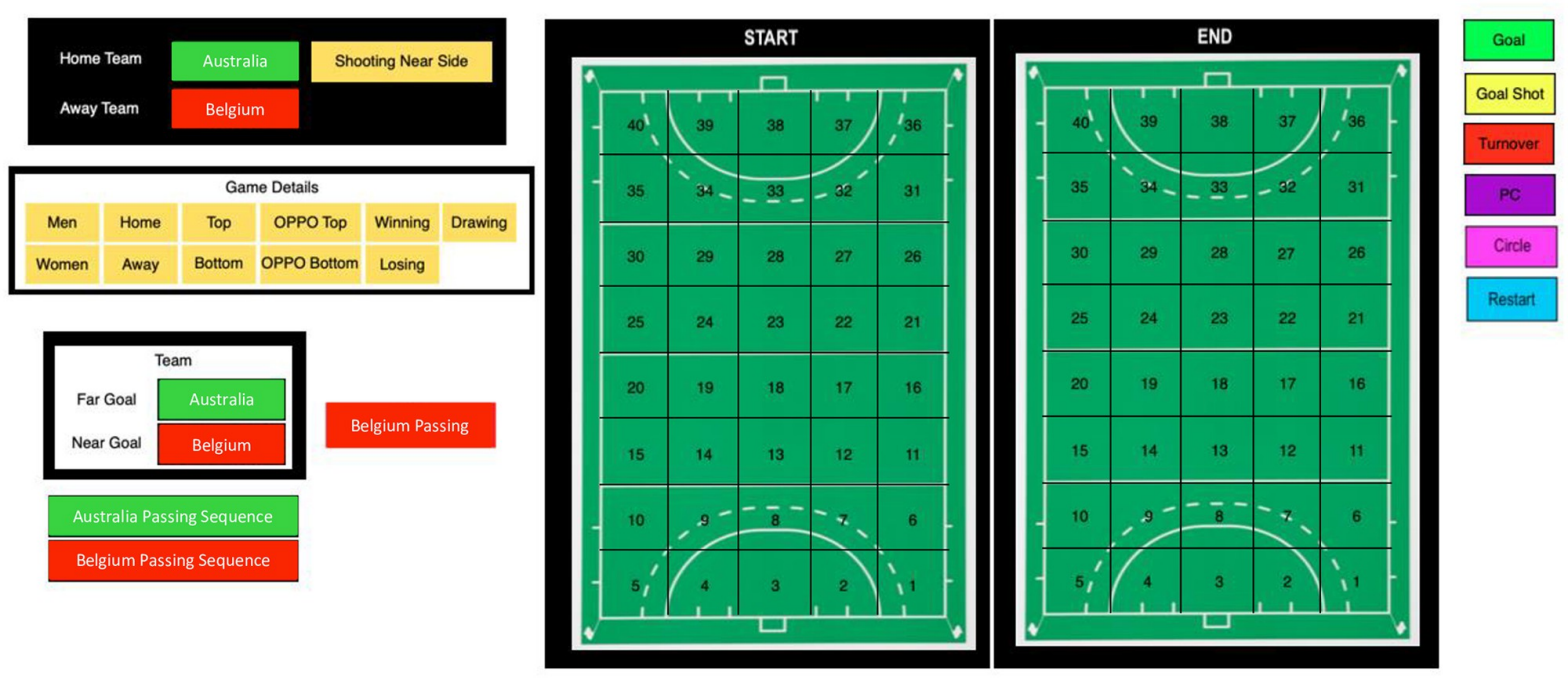
Notational analysis system used to capture ball movement patterns in SportsCode. Example shows a field hockey game between Australia and Belgium. An analyst would choose the team in possession, the start and end location of each ball movement followed by the outcome at the end of a play. The field orientation would switch direction depending on which team was in possession so that cell 1 always corresponded to the attacking 25 left corner and cell 40 to the defensive 25 right corner of the field.

The code window for tracking ball movement is illustrated in Fig 4. This window represents a field divided into 40 zones of equal size. Code rows included each individual ball movement and each possession sequence, and start and end locations, play outcomes and match context labelled in each code row. This structure allowed overall ball movements or sequences of play to be analysed in relation to outcomes. The XML file for both in-game events and ball movements were then exported from SportsCode^®^, and then imported into Microsoft Excel^®^ so that data could be analysed in another programme. The XML file (Fig 2B) contains all information recorded in chronological order, where each row of data represents an individual instance from a code row with descriptive labels and time stamp, indicating start and end times of the event, included. Raw data is available here, data collection and analysis methods comply with the terms and conditions of the source of the data.

### Data analysis

Data analysis was completed in RStudio 1.3.1093 (RStudio Inc, https://www.rstudio.com), a statistical programming integrated development environment, using the R (version 4.03) programming language. The R code detailing the instructions on how to analyse game styles, ball movements and in-game events is available here. Alternatively, the R code and output is available at their respective hyperlinks (game styles, ball movements, in-game events) so that instructions can be viewed without access to RStudio. Each team per match per match status period (winning, losing, and drawing) were analysed separately to identify strategies used by individual teams under different match conditions. Attack profiles were produced when a team was in attack and defence profiles produced by analysing opposition values.

### Game styles

Refer to Lord et al., [23] for a detailed explanation of chosen variables and data analysis methods. Normalised variables (calculated from their code row) included in the creation of game styles were:

- Game Actions: game action types per location per attack type (%), ratio of movement effects per location per attack type, time per attack type per location, game action totals per attack type
- Stoppages: stoppages per location (%), stoppages per attack type (%), restart speed ratios per location per attack type, set pieces per attack type (%)
- Turnovers: turnovers per attack type (%), turnover types per attack type (%), turnover locations per attack type (%), turnover pressures per attack type (%)
- Goal shots: goal shots per attack types, goals per attack type
- Attack type: number per game (established attack, counter attack, set piece)

To identify game styles in this study, 102 dynamic game variables were divided into 6 predetermined game style categories which consisted of: 1) Established Attack Game Actions, 2) Counter Attack Game Actions, 3) Established Attack Success, 4) Counter Attack Success, 5) Set Piece Occurrence, and 6) Tempo. A k-means cluster analysis, an unsupervised classification technique, was performed on each category using the ‘*kmeans’* function from the *‘stats’* R package [36]. The number of clusters chosen should be the largest amount that reduces the variation between clusters, and reflects the common strategies or outcomes in a category of performance.

For this study, 2 clusters were selected per game style category and practical identities given to each that described the different approaches so strategy could be easily communicated. Game style types included strong or poor for established and counter attack success, high or low for set piece occurrence, pass or dribble for established and counter attack game actions and direct or possession for tempo. Bootstrapping, using the *‘clusterboot’* function from the *‘fpc’* package [37] was undertaken to assess reliability, with >75% of iterations produced consistently. A game style profile was produced by calculating the percentage of games within each game style type. This process reliably reduced the data from 102 dynamic game variables to 6 key categories of performance that reflect how a team plays and areas of strength and weakness.

### Ball movements

**Fig 5:**
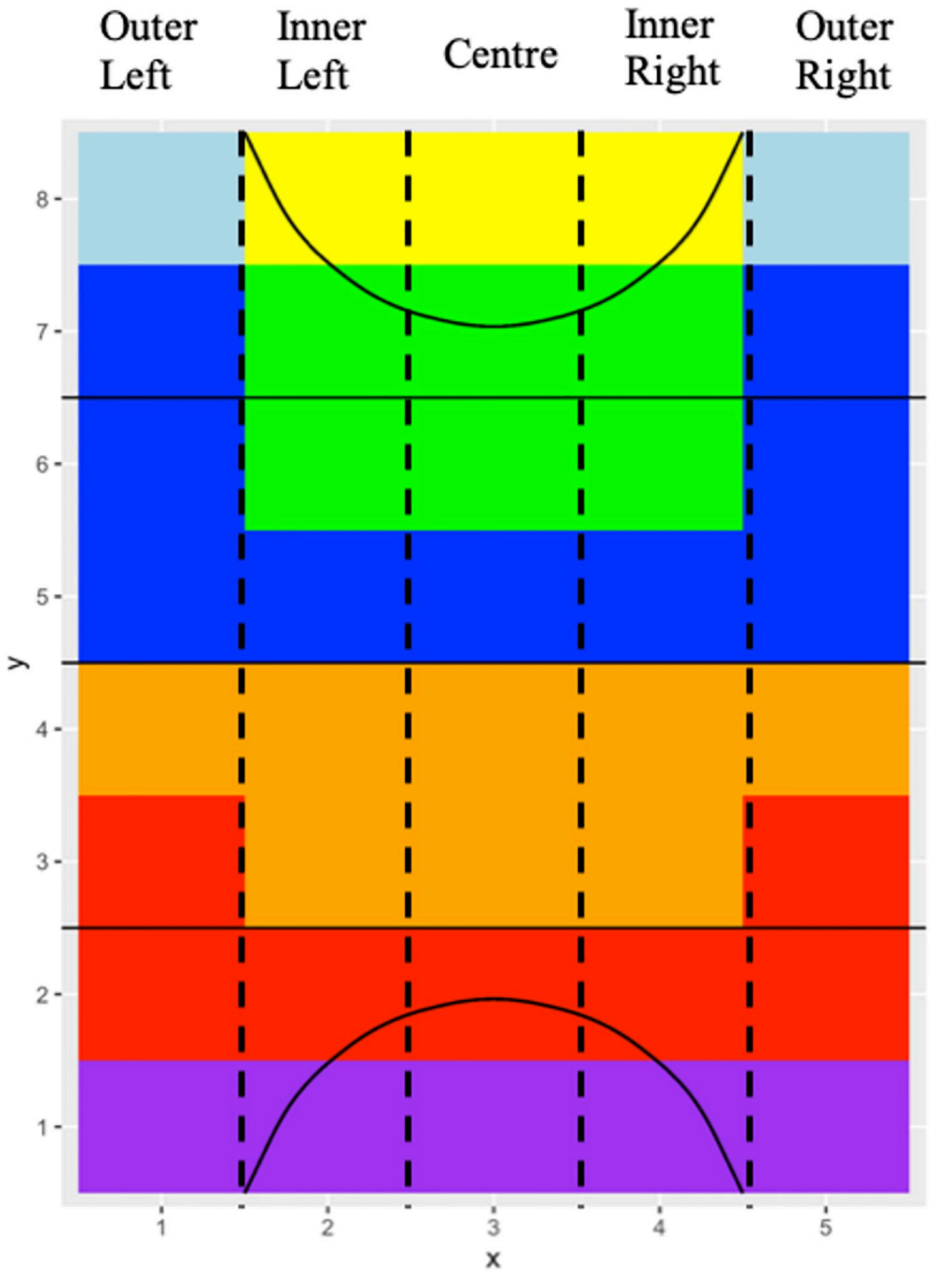
Field hockey pitch divided into seven attacking zones. Yellow = circle, light blue = corners, green = deep attack, dark blue = build attack, orange = build defence, red = outlet, purple = deep defence.

The information gained from ball tracking data was converted from locations on the field to represent where a team had possession, how unpredictable (entropy) a team was in their movements, and the direction of ball movements in different areas of the field. Data was simplified from 40 cells into 7 attacking zones, which are illustrated in Fig 5, to represent different phases of play. The variables analysed for each attacking zone included:

- Possession—the time to complete ball movements
- Standardised Entropy—a measure of unpredictability of a ball movement from one cell to all other cells on the field
- Progression rates—the percentage of ball movements in each direction between attack zones; back (end zone behind start zone), stay (start and end zones are the same), forward (end zone 1 ahead of start zone in the defensive half, or 1 ahead but not direct to goal in the attacking half), goal (end zone 2 ahead from start zone from defensive half or 1 ahead, but direct through centre of the field to goal from the attacking half)
- Game possession—the total number of ball movements in all attacking zones between teams for each match

### In game events

The data capture process in this study allowed further analysis of in-game events including:

- Movement effects per game action type per attack type
- Goal shot locations, outcomes, and efficiencies
- Penalty corner routines and outcomes
- Stoppage types per location
- Turnover types per location
- Possession time, length, and rate
- Starting locations per attack types
- End locations per start location per attack types

These variables were excluded from the game styles analysis given a lack of variation between game style types however individual team differences can be observed. Data analysis involved calculating percentages for each outcome or type of action. Including a separate code row for each variable for analysis, permitted simple extraction of the required information, calculation of totals by counting the number of instances within a code row, and subsequent conversion to percentages.

### Data visualisation and communication

Data visualisation was completed in RStudio to create an interactive application, called a Shiny app, to aggregate all data analysis into one product. A Shiny app, created using the ‘*shiny*’ R package [38], is composed of a user interface (ui) file which controls the design and appearance of the app, and a server file which contains the instructions to generate the visualisations. The final product is an interactive web application which allows a user (such as a coach or player) to view the results on a web browser, without needing specific software or expertise. The ui and server files, illustrating the design and creation of figures, are detailed here.

**Fig 6:**
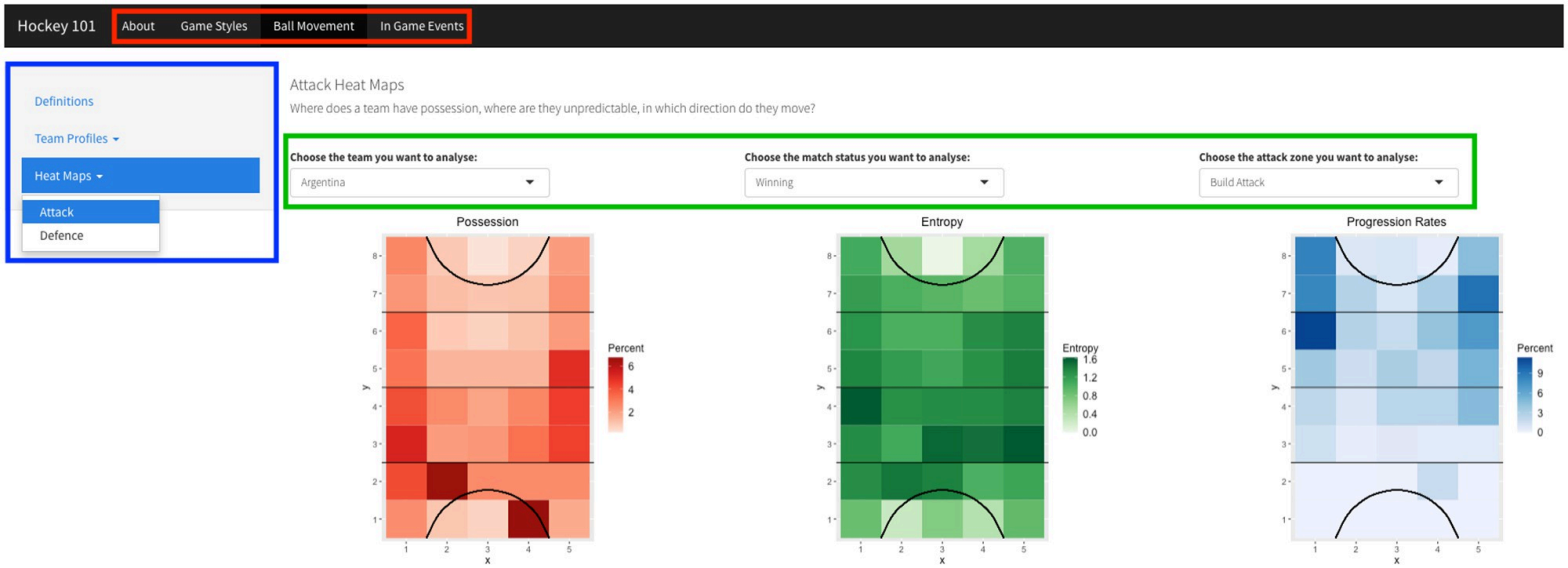
Shiny application layout. Tabs are outlined in red, side panel and subcategory list in blue and filter options in green above the visualisation.

Fig 6 illustrates the design of the app. The application is divided into 4 tabs at the top of the page using the ‘*navbarPage’* function including a page describing how to use and interpret the results, and then separate tabs for game styles, ball movements and in-game events. Within each tab, a list of sub-categories of visualisations are presented, using the ‘*navlistPanel’* function, along the left-hand side as a side panel. Each sub-category contains a list of figures available to analyse for that topic using the ‘*navbarMenu’* function. A practical feature of Shiny apps is the ability to interact with the data using ‘*reactive’* functions. These functions filter the data to the chosen criteria which automatically updates the figures. Consequently, multiple figures are not needed to be produced per team or context as an individual figure can easily be manipulated to compare different situations. The main panel of the app displays the visualisation and filter options available. For example, in this app, a coach can choose the team, match status, field location or attack type to analyse.

Team data is presented as an average in comparison to the league average so that strengths and weaknesses, and similarities and differences, can be identified. Z-scores (normalised standard deviation from the mean) are also used to reflect the magnitude of differences when comparing variables on different scales. All figures are produced using the ‘*ggplot’* function from the *‘ggplot2’* R package [39]. Within this function the type of plot produced must be specified. Tile plots and heat maps are produced using ‘*geom_tile’*, columns charts with ‘*geom_col’* and scatterplots with ‘*geom_point’*. To display the visualisation in the app, the *‘renderPlot’* function is specified in the ui file.

## Results

**Fig 7:**
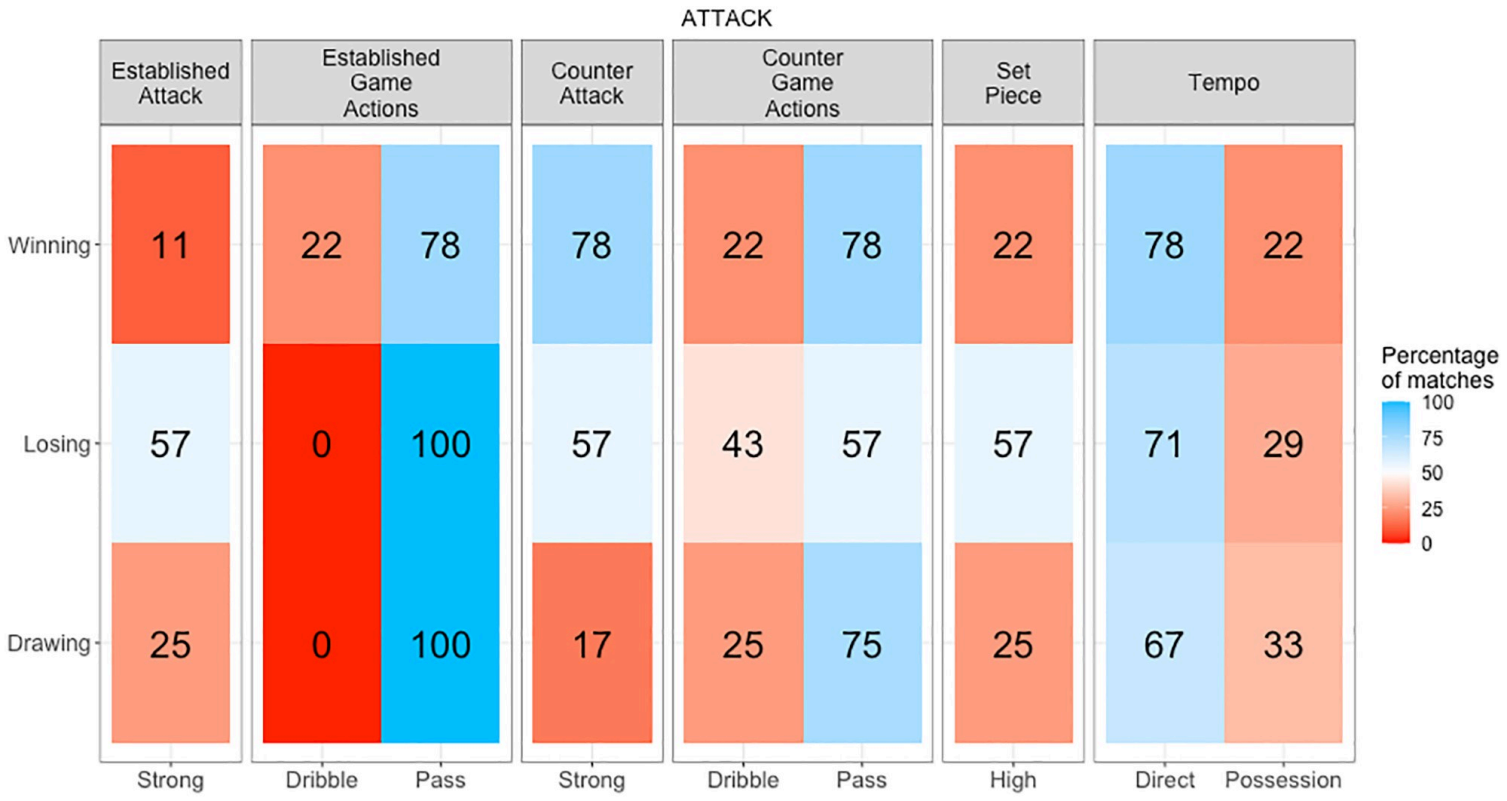
Attack game style profile. Percentage of matches in each game style type grouped by game style category on the x axis per match status on the y axis. Red shading indicates a game style used rarely, white an inconsistent game style and blue a consistent game style.

**Fig 8:**
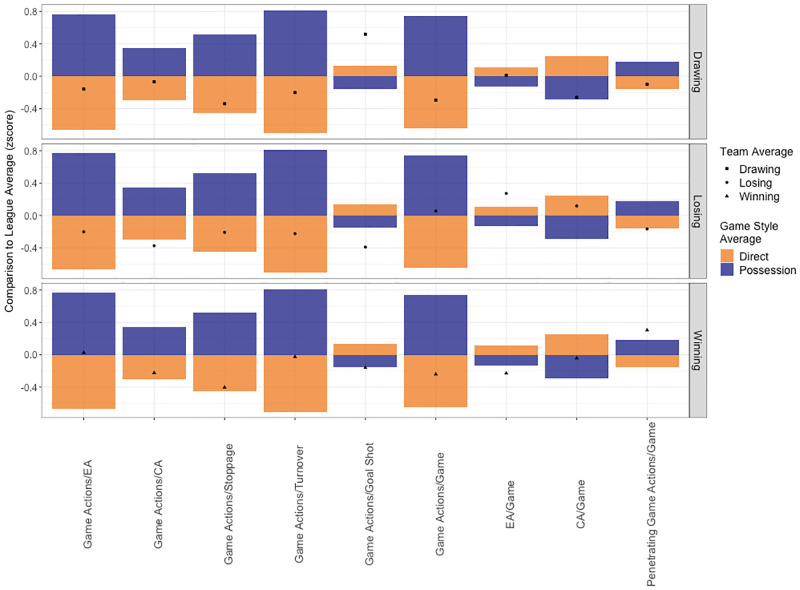
Variables included in the tempo game style category. Game variables are presented on the x axis and z-scores per match status on the y axis. Bars represent average values for each game style type (direct or possession) and shapes represent team averages per match status. A team average of 0 indicates an inconsistent variable for that game style as it does not strongly reflect either game style type, for example Game Actions/EA when winning. A team average within the game style bar reflects variables reflecting their game style type, for example Game Actions/Stoppage. A team average that is greater than the game style average indicates a variable strongly related to the game style type, for example Game Actions/Goal Shot when drawing. A team average that is within the opposite game style type bar reflects a variable differentiating the team from teams using the same game style type, for example EA/Game when winning is classed as a possession characteristic but the team is classed as a direct game style overall.

**Fig 9:**
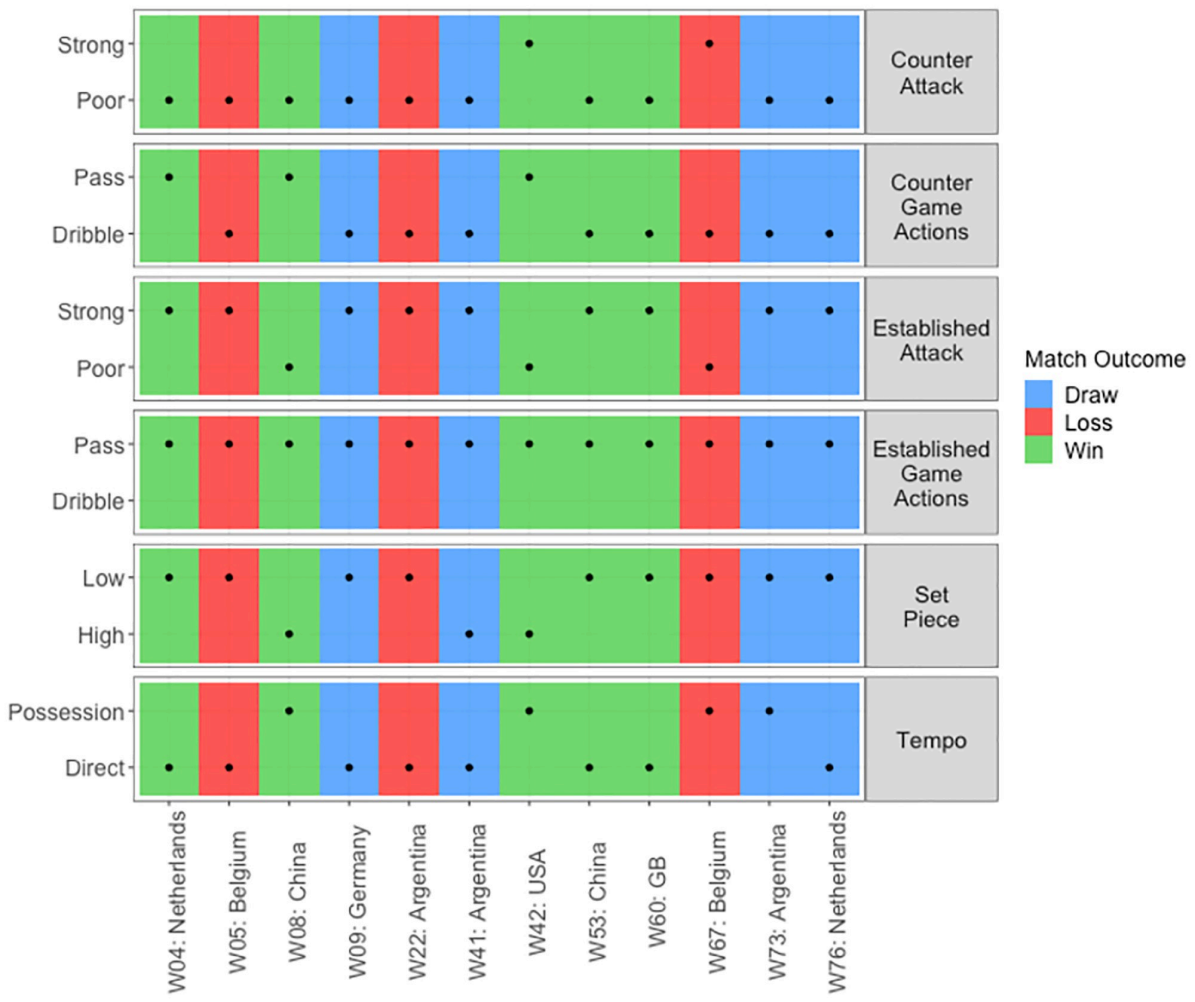
Game style types per match outcomes. Match per opponent are presented on the x axis and game style types per game style category on the y axis. Background colours indicate match outcome, blue indicates a draw, red a loss and green a win. The game style type used per match is indicated by a black dot.

**Fig 10:**
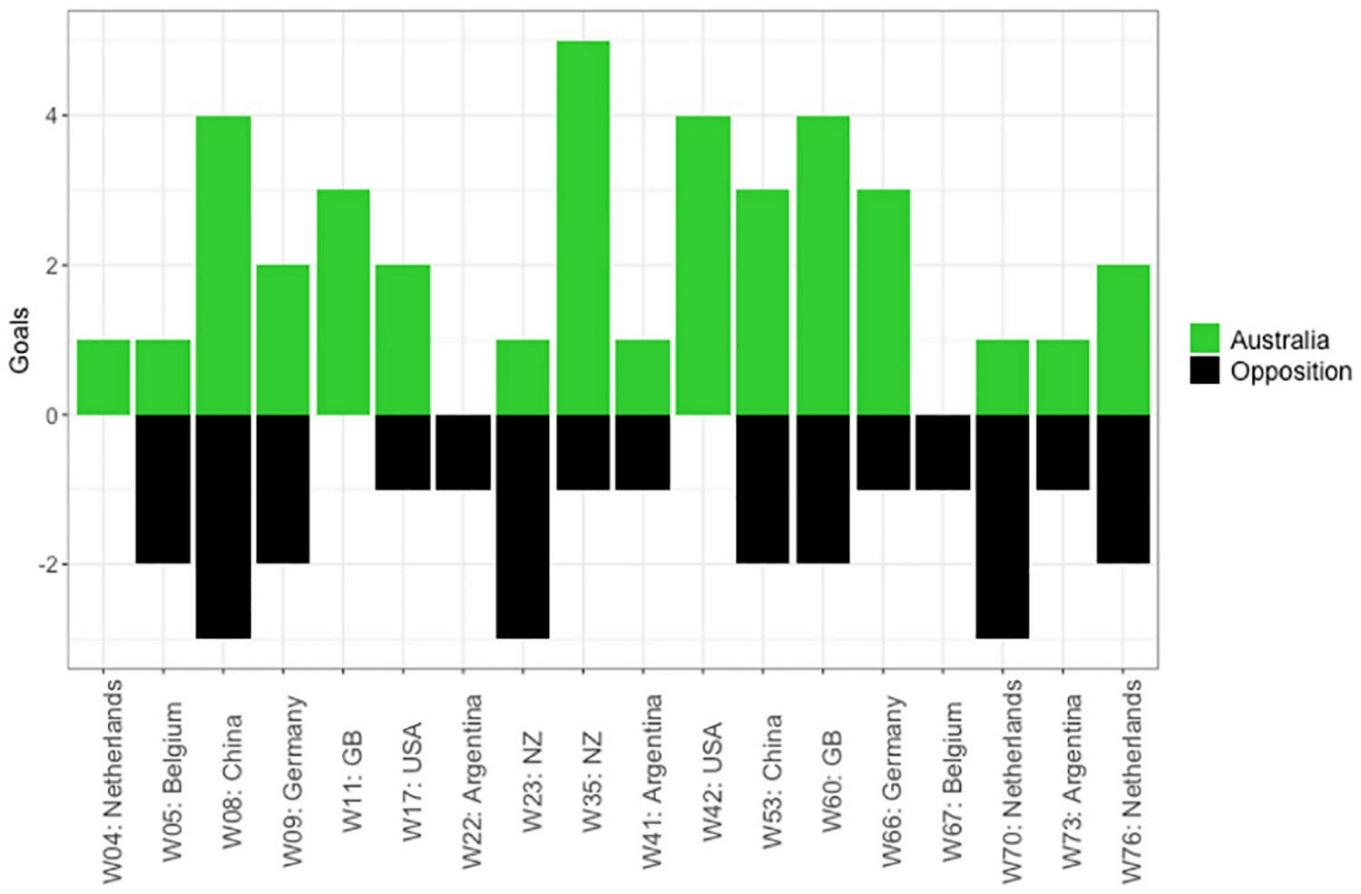
Goals for and against per match. Match per opposition are presented on the x axis, and goals for the reference team on the positive y axis in green and goals for the opposition on the negative y axis in black.

**Fig 11:**
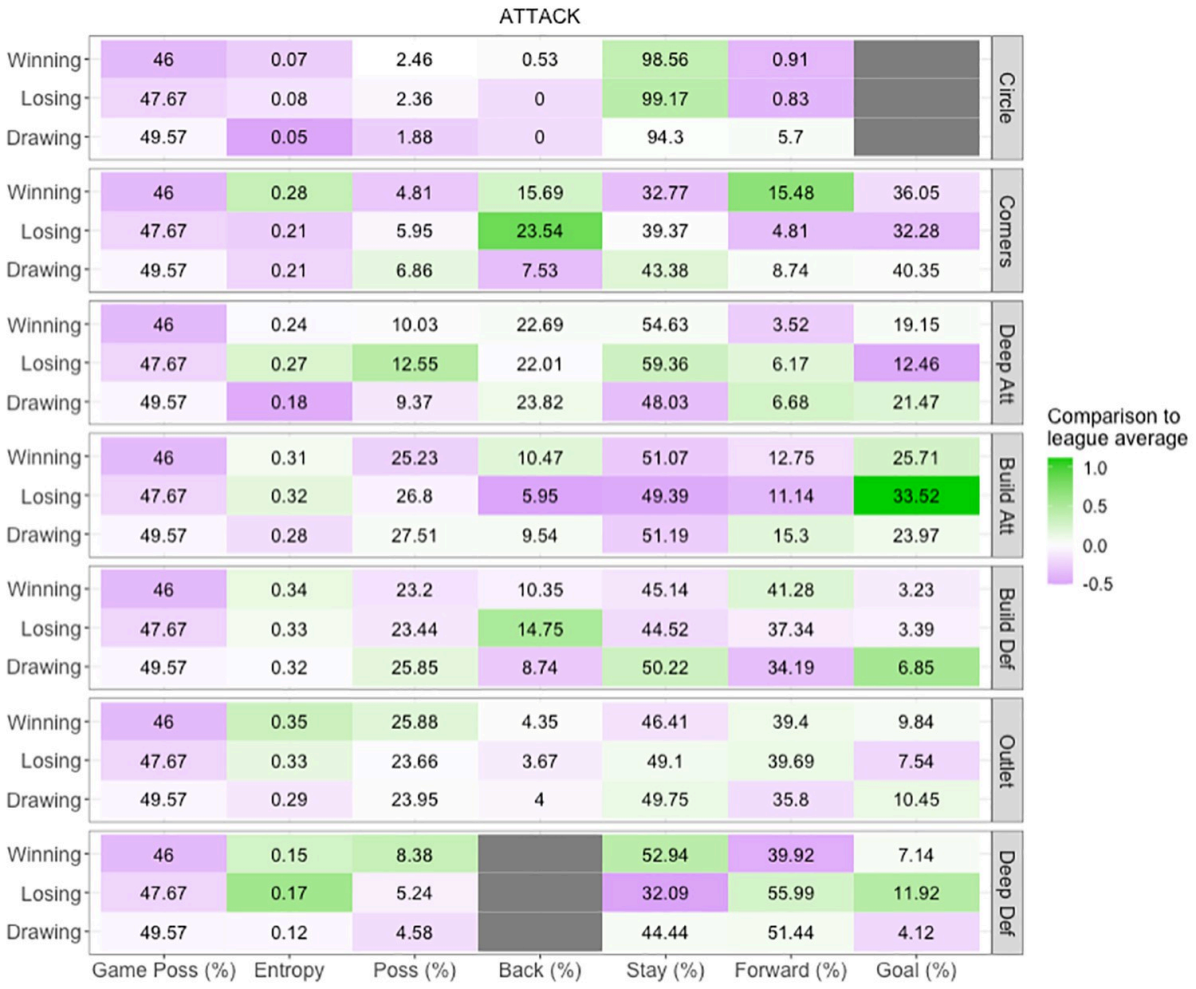
Ball movement profile. Ball movement variable on the x axis per match status per attacking zone on the y axis. Colour scale represents z-scores, purple shading indicates variables that are below average, white average and green above average performance compared to the league.

**Fig 12:**
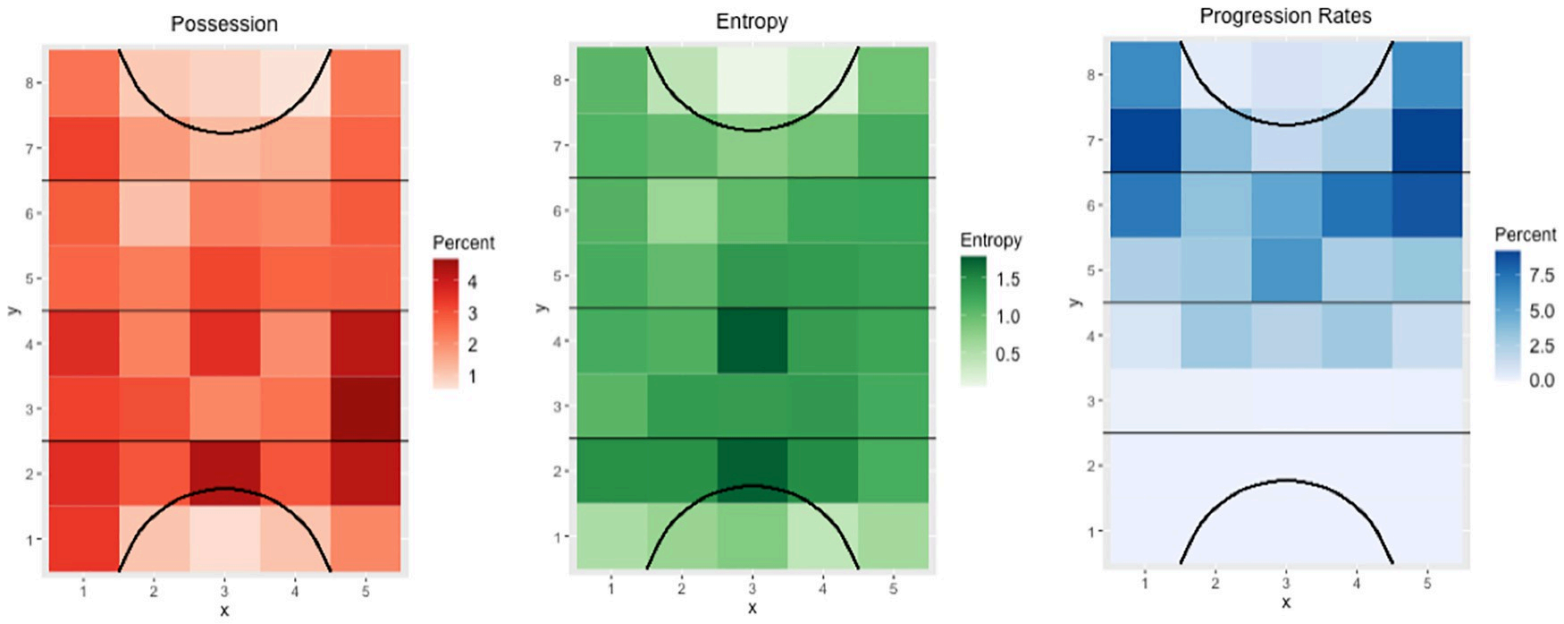
Heat maps illustrating possession (left), entropy (middle) and progression rates (right). Teams attack towards the top of the figure. Light to dark colour scales indicate low to high levels of the variable. Each x/y coordinate represents one cell from the data capture process.

**Fig 13:**
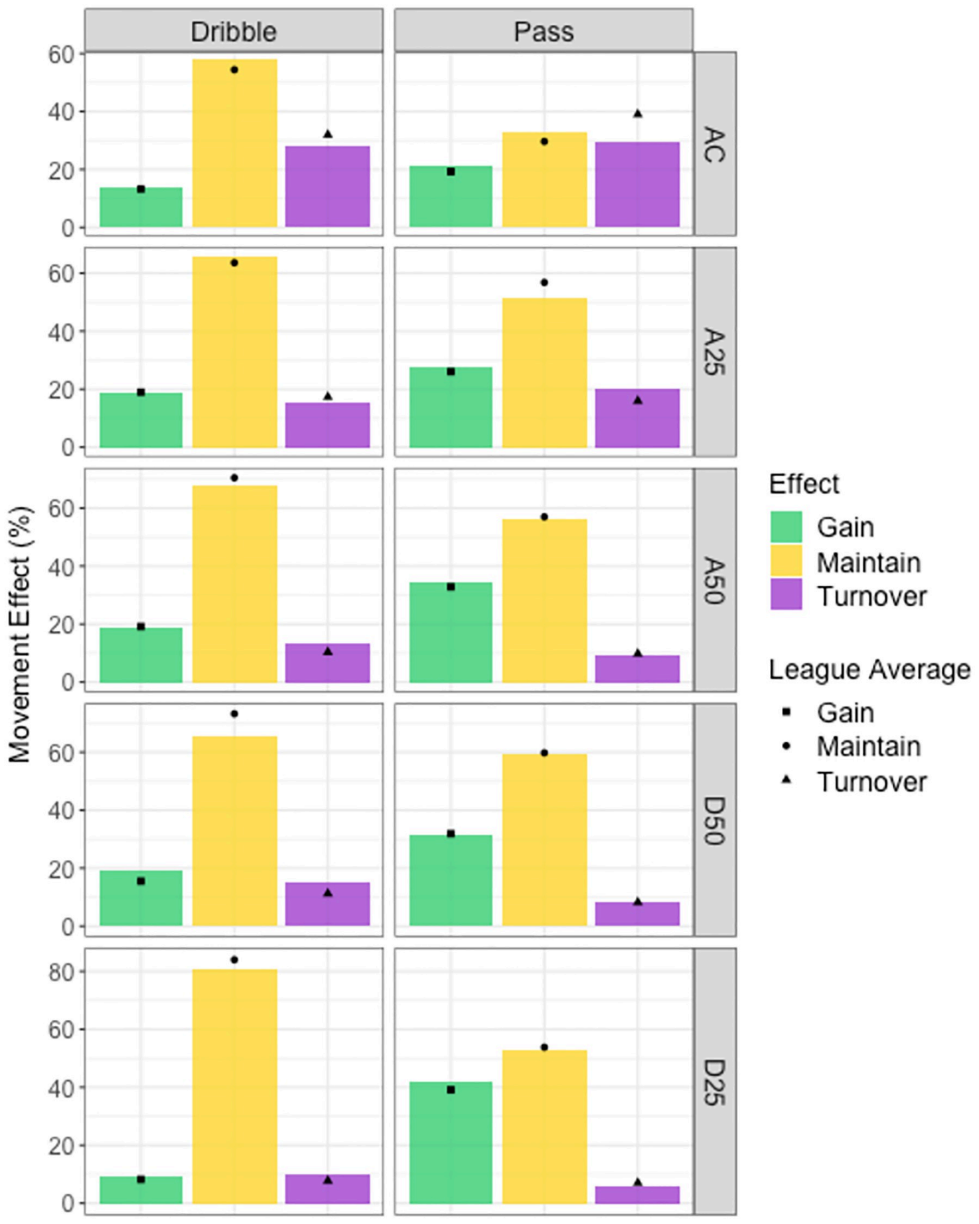
An example in-game events visualisation. Dribble and pass game actions on the x axis per movement effect per field location on the y axis. Coloured bars indicate team average per movement effect, and shapes indicate league average per movement effect. Team performance is greater than the league when the bar is greater than the shape, and less than the league when the bar is lower than the shape. For example, this team is less likely to turn over the ball when dribbling in AC (attacking circle).

**Fig 14:**
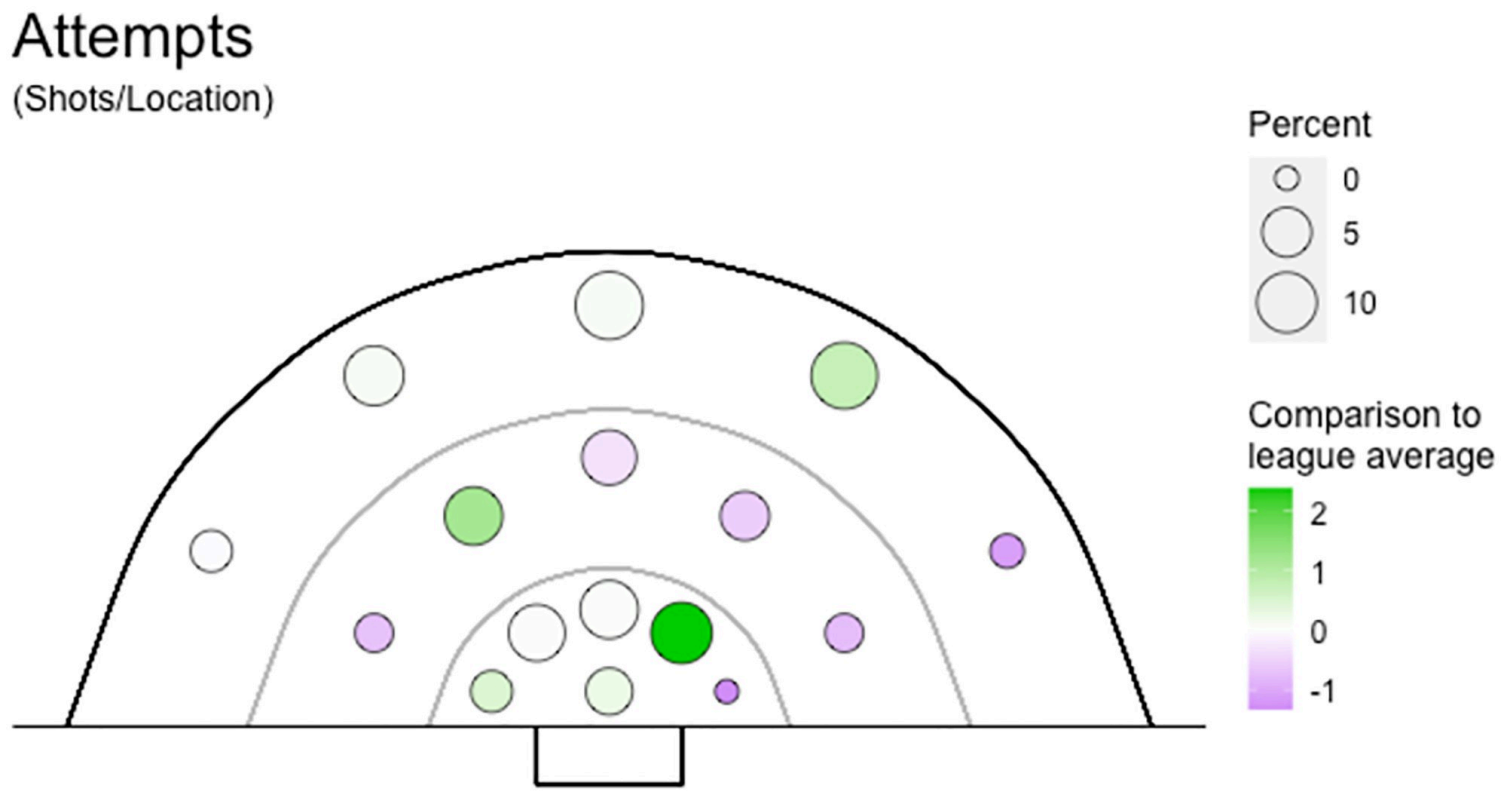
Goal shots heat map. The percentage of goal shots from each of the 16 shooting locations for one team is illustrated. The size of the circle indicates the percentage of shots from that location, with increasing size indicating a greater percentage of shots. Colour indicates comparison to the league average, purple indicating below average and green above average performance. For example, this team has approximately 10% of shots from the middle inner right zone (from the perspective of the shooter looking at the goals) which is a greater percentage than the league average.

Access to the application created to visualise data analysing strategy in hockey is available at this hyperlink. The Shiny app is deployed using an external server hosted by RStudio (https://shinyapps.io) which allows users to view and interact with the application without needing access to RStudio. Examples of each type of visualisation are provided to illustrate how figure types and aesthetics have been used to communicate different parts of strategy. Fig 7 illustrates an example of a game style profile, Fig 8 shows an example of a game style categories variables, Fig 9 illustrates game style types per match outcome, Fig 10 displays goals for and against per match, Fig 11 illustrates a ball movement profile, Fig 12 displays heat maps for possession, entropy and progression rates, Fig 13 details an example in-game event profile for movement effects per game action and Fig 14 shows a goal shot map.

### Data interpretation

The app produces layers of simple to detailed visualisations that provide insight into understanding, developing and communicating strategy for individual teams in different match situations. The first layer of analysis provides insight into the holistic game plan and reflects key attributes of the game a coach can observe and communicate (Figs 7 and 11). The second level of analysis identifies differences and weaknesses in a team’s strategy by breaking down the key attributes into their contributing parts, and understanding how the holistic strategy is achieved (Figs 8–10 and 12). The third level of analysis identifies ways of exploiting opposition weaknesses and inhibiting opposition strengths, and provides the detail to develop strategy by linking the techno-tactical indicators with the holistic game plan (Figs 13 and 14). The process to analyse strategy is outlined by describing the information that can be gained from each visualisation, and how it relates to strategy development through interpreting hypothetical scenarios.

The initial visualisation to analyse is a game style profile (Fig 7). Game style profiles allow easy communication of strategy as they reflect the key attributes a team consistently implements in practical terms. The key aspects of performance include how a team moves the ball, how strong they are in different attacks, and their ability or intent to control the ball. These form the basic elements of all team invasion sports, and their interactions describe the strategies implemented by teams. Common strategies emerge from analysing these interactions. For example, hockey teams who are strong in established attacks using a possession approach like to control the game. However, teams who are strong in counter attack and play direct, prefer attacking quickly while the opposition is unbalanced. Alternatively, a team who are strong in established attack, but poor in established defence, creates and concedes high attacking opportunities by playing a high-risk game to outscore the opponent. In contrast, a team who are poor in established attack, but strong in established defence, creates and concedes few attacking opportunities by playing a safer strategy prioritising defence over attack.

After identifying a team’s game style type for each category of performance, an analyst can dive deeper into the variables relating to each game style type to understand why a team is classified this way (Fig 8). This second layer of analysis evaluates the game variables that generate a game style type, and identifies the individual nuances separating teams with similar game styles. If a team average is equal to or above the league average for a game variable within their game style type, this style should be monitored during a game to identify if a team is executing their preferred strategy. However, a team identifying with a variable in the opposite game style type indicates a difference in their strategy compared to other teams. Furthermore, if a team average is centred between game style types it indicates a variable that is inconsistent between matches. For example, a team may be classed as using a direct game style, typically classified by fewer game actions per game and possession but a higher number of established and counter attacks per game. However, this team records fewer counter attacks per game (a variable associated with a possession game style) indicating that although they want to attack directly, as they may not have the skills to control the ball for long periods of time, attacking with a balanced line up is preferred. This scenario places the team in a better position defensively as they are more likely to have greater numbers around the ball when they do turn it over.

An analyst may then evaluate game styles per match (Fig 9) and identify effective strategies each team used by relating match status and match outcomes. For example, game style types used during periods when a team were drawing could be deemed successful if this team consequently went on to win the match. However, if a team were losing and eventually lost the match, these tactics were not effective. Additionally, analysing goal differences (Fig 10) in relation to game styles per match outcome indicates the effectiveness of the strategies implemented, and the intent of teams to continue attacking or maintain a lead. For example, teams that consistently win low scoring games may be strong defensively, but average offensively, so they will look to protect a lead if they get ahead. Comparatively, a team that win games by a large margin may be strong in both attack and defence, so they will continue to seek goal scoring opportunities.

Ball movement profiles (Fig 11) provide a complementary insight into a team’s game style, providing easy-to-communicate analysis by simplifying ball tracking information into practical descriptors. These profiles can further describe how a team moves the ball from a spatial perspective by analysing how much game control a team has, the predictability and direction of this movement, and the length of possession and opportunities within an attacking zone. Common ball movement strategies can also be identified; for example, a team with high entropy and possession in the attacking half with average progression rates attempt to control possession by utilising the length and width of the field. In contrast, a team with low entropy but high possession and forward movements in the attacking half can create a greater number of opportunities at goal by playing quickly and directly in attack.

By understanding the ball movement style, an analyst can then observe specific areas of the field using heat maps (Fig 12). Heat maps allow easy interpretation of key areas on the field to provide insight into whether teams are more likely to use one side of the field, the inner or outer channels or through the centre. This second layer analysis also indicates how a team is structured in different phases of the game allowing specific plays to be developed to counteract the oppositions likely movements. For example, a team that has a higher percentage of possession in build defence on the outer and centre channels indicates the team likely plays with a back three, as opposed to higher possession in the inner channels evidence of a team playing with two central and two wing defenders.

In-game descriptive events from game styles analysis and spatial data from ball movement patterns can be linked to help explain differences in strategy. For example, a team can be strong in established attack as they have greater actions and events in the attacking half, however they create fewer goal shots than average for this game style type. By observing ball movement profiles, it can be identified this team has greater possession in the build attack zone and corners, with heat maps illustrating a greater likelihood of transferring the ball around the midfield and through the left corner to enter the circle. This pattern allows the opposition to prevent goal shots by setting up defensively in advantageous positions to goal. The interaction of these two types of analysis allows the *how* and *where* information of ball movement to be linked to provide greater insight into a team’s success.

Studying in-game events provides a third layer of analysis on specific phases or events during a game, and breaks down the components a strategy is built around (Figs 13 and 14). This approach provides insight into how to exploit weaknesses or differences identified in game style or ball movement variables. This third level of analysis provides meaning behind the strategy rather than simply identifying the underlying team strategy. Understanding the overall plan for a team makes it is easier to understand how each component relates to this strategy. This is certainly the case compared to trying to understand a collection of detailed instructions without knowing the desired outcome. For example, (1) game style analysis reveals a team is strong in established attacks and prefers dribbling, (2) analysis of game variables of the established attack game style category shows this team records a higher percentage of penalty corners and less goal shots, (3) analysing in-game events identifies a team is more likely to maintain possession when dribbling, than gain ground, and have a higher percentage of stoppages as free hits in the attacking 25. These three layers of analysis reflect a team strategy that aims to control possession, rather than attack the goals, and engage in individual contests to win free hits. Defensively to counteract this strategy, a coach would select players with high tackling ability and who can implement a zone defence, rather than man-on-man marking, to force the opposition to play around them rather than through them, to limit their opportunities for penalty corners as their preferred goal scoring method.

As another example, a team is identified as having lower possession, lower entropy and higher forward movements in the build defence zone. Heat maps can be used to reveal this team is more likely to move the ball down the outer edges of the field using long passes. Analysis of in-game events identifies this team has a greater percentage of turnovers from intercepts and out of bounds. Therefore, this team’s strategy is to attack quickly but predictably in the forward direction, and risk long passes to get behind lines of the defence into space. A coach can instruct their forwards and midfield to have high pressure on the ball carrier, and their defenders to play in front of their opposition strikers, and protect the dangerous passing channels. These tactics should force the opposition to throw riskier long passes with a greater chance of a turnover occurring, or pass short or dribble through the opposition which does not suit their abilities.

## Conclusion

We have outlined a practical approach to performance analysis in team invasion sports by identifying the key steps when capturing, analysing, visualising, and communicating data to gain insight into a team’s strategy. Completing each step with a practical focus yields important insights that can directly inform a team’s strategy. An example was provided analysing strategy in field hockey, and illustrated the key themes captured, methods of data analysis to extract layers of effective insights, and development of an interactive application for easy access and communication of visualisations. This performance analysis process can be applied to any team invasion sport providing practical insights for coaches to develop and communicate specific strategies for different match situations.

## Supporting information

S1 File (DOCX)

## References

[1] Martin D, O’Donoghue PG, Bradley J, McGrath D. Developing a framework for professional practice in applied performance analysis. Int J of Perform Anal Sport. 2021;21: 845–888.

[2] Espenschade A. An analysis of activity records of field hockey players. Res Q Am Phys Educ Assoc. 1936;7: 62–74.

[3] Lord F, Pyne DB, Welvaert M, Mara JK. Methods of performance analysis in team invasion sports: A systematic review. J Sports Sci. 2020;38: 2338–2349. doi: 10.1080/02640414.2020.1785185 32583724

[4] Brouner J. Performance analysis–Supporting coach feedback. In: Gill AJG, editors. Foundations of Sports Coaching. Routledge; 2021. pp. 179–197.

[5] Wright C, Atkins S, Jones B, Todd J. The role of performance analysts within the coaching process: Performance Analysts Survey ‘The role of performance analysts in elite football club settings.’. Int J Perform Anal Sport. 2013;13: 240–261.

[6] Ribeiro J, Davids K, Araújo D, Silva P, Ramos J, Lopes R et al. The role of hypernetworks as a multilevel methodology for modelling and understanding dynamics of team sports performance. Sports Med. 2019;49: 1337–1344. doi: 10.1007/s40279-019-01104-x 31016547

[7] Ribeiro J, Silva P, Duarte R, Davids K, Garganta J. Team sports performance analysed through the lens of social network theory: implications for research and practice. Sports Med. 2017;47: 1689–1696. doi: 10.1007/s40279-017-0695-1 28197801

[8] Soltanzadeh S, Mooney M. Systems thinking and team performance analysis. Int Sport Coach J. 2016;3: 184–191.

[9] Travassos B, Davids K, Araujo D, Pedro Esteves T. Performance analysis in team sports: Advances from an Ecological Dynamics approach. Int J Perform Anal Sport. 2013;13: 83–95.

[10] Salmon PM, McLean S. Complexity in the beautiful game: implications for football research and practice. Sci Med Footb. 2020;4: 162–167.

[11] Barris S, Button C. A review of vision-based motion analysis in sport. Sports Med. 2008;38: 1025–1043. doi: 10.2165/00007256-200838120-00006 19026019

[12] Robertson S. Man & machine: Adaptive tools for the contemporary performance analyst. J Sports Sci. 2020;38: 2118–2126. doi: 10.1080/02640414.2020.1774143 32530736

[13] Ortega-Toro E, Garcia-Angulo A, Gimenez-Egido JM, Garcia-Angulo FJ, Palao JM. Design, validation, and reliability of an observation instrument for technical and tactical actions of the offense phase in soccer. Front Psychol. 2019;10: 22. doi: 10.3389/fpsyg.2019.00022 30733691 PMC6353797

[14] Villarejo D, Ortega E, Gomez M-A, Palao J-M. Design, validation, and reliability of an observational instrument for ball possessions in rugby union. Int J Perform Anal Sport. 2014;14: 955–967.

[15] Gollan S, Ferrar K, Norton K. Characterising game styles in the English Premier League using the "moments of play" framework. Int J Perform Anal Sport. 2018;18: 998–1009.

[16] Fernandez-Navarro J, Fradua L, Zubillaga A, Ford PR, McRobert AP. Attacking and defensive styles of play in soccer: analysis of Spanish and English elite teams. J Sports Sci. 2016;34: 2195–2204. doi: 10.1080/02640414.2016.1169309 27052355

[17] Lago-Penas C, Gomez-Ruano M, Yang G. Styles of play in professional soccer: an approach of the Chinese Soccer Super League. Int J Perform Anal Sport. 2017;17: 1073–1084.

[18] Losada AG, Theron R, Benito A. BKViz: A basketball visual analysis tool. IEEE Comput Graph Appl. 2016;36: 58–68. doi: 10.1109/MCG.2016.124 27893368

[19] Perin C, Vuillemot R, Fekete J-D. SoccerStories: A kick-off for visual soccer analysis. IEEE Trans Vis Comput Graph. 2013;19: 2506–2515. doi: 10.1109/TVCG.2013.192 24051817

[20] Polk T, Yang J, Hu Y, Zhao Y. Tennivis: Visualization for tennis match analysis. IEEE Trans Vis Comput Graph. 2014;20: 2339–2348. doi: 10.1109/TVCG.2014.2346445 26356948

[21] O’Donoghue P. Reliability issues in performance analysis. Int J Perform Anal Sport. 2007;7: 35–48.

[22] Hughes MD, Bartlett RM. The use of performance indicators in performance analysis. J Sports Sci. 2002;20: 739–754. doi: 10.1080/026404102320675602 12363292

[23] Lord F, Pyne DB, Welvaert M, Mara JK. Identifying and analysing game styles and factors influencing a team’s strategy in field hockey. J Sports Sci. 2022;40: 908–919. doi: 10.1080/02640414.2022.2037839 35139755

[24] Castellano J, Pic M. Identification and preference of game styles in LaLiga associated with match outcomes. Int J Environ Res Public Health. 2019;16: 5090. doi: 10.3390/ijerph16245090 31847147 PMC6950299

[25] Hewitt A, Greenham G, Norton K. Game style in soccer: what is it and can we quantify it? Int J Perform Anal Sport. 2016;16: 355–372.

[26] Greenham G, Hewitt A, Norton K. A pilot study to measure game style within Australian football. Int J Perform Anal Sport. 2017;17: 576–585.

[27] Schelling X, Robertson S. A development framework for decision support systems in high-performance sport. Int J Comput Sci Sport. 2020;19: 1–23.

[28] Kale A, Nguyen F, Kay M, Hullman J. Hypothetical outcome plots help untrained observers judge trends in ambiguous data. IEEE Trans Vis Comput Graph. 2018;25: 892–902. doi: 10.1109/TVCG.2018.2864909 30136961

[29] Midway SR. Principles of effective data visualization. Patterns. 2020;1: 100141. doi: 10.1016/j.patter.2020.100141 33336199 PMC7733875

[30] Cawthon N, Moere AV. The effect of aesthetic on the usability of data visualization. 2007 11th International Conference Information Visualization (IV’07). 2007 4–6 Jul; Zurich, Switzerland, p. 637–48.

[31] Weissgerber TL, Winham SJ, Heinzen EP, Milin-Lazovic J, Garcia-Valencia O, Bukumiric Z et al. Reveal, don’t conceal: transforming data visualization to improve transparency. Circulation. 2019;140: 1506–1518. doi: 10.1161/CIRCULATIONAHA.118.037777 31657957 PMC8947810

[32] Evergreen S, Metzner C. Design principles for data visualization in evaluation. In: Azzam T, Evergreen S, editors. Data Visulization part 2. New Directions for Evaluation; 2013. pp. 5–20.

[33] Joyce SC. Web based data visualisation applied to creative decision making in parametric structural design. Proceedings of IASS Annual Symposia; 2015; International Association for Shell and Spatial Structures (IASS). p. 1–12.

[34] Yi JS, ah Kang Y, Stasko J, Jacko JA. Toward a deeper understanding of the role of interaction in information visualization. IEEE Trans Vis Comput Graph. 2007;13: 1224–1231. doi: 10.1109/TVCG.2007.70515 17968068

[35] Altman DG. Practical statistics for medical research. London: Chapman & Hall; 1991. pp. 404

[36] R Core Team (2020). R: A language and environment for statistical computing. R Foundation for Statistical Computing, Vienna, Austria. https://www.R-project.org/

[37] Hennig C. (2020) fpc: Flexible Procedures for Clustering. R package version 2.2–9. https://CRAN.R-project.org/package=fpc

[38] Chang W, Cheng J, Allaire JJ, Sievert C, Schloerke B, Xie Y, et al. (2021). shiny: Web Application Framework for R. R package version 1.6.0. https://CRAN.R-project.org/package=shiny

[39] Wickham H. ggplot2: Elegant Graphics for Data Analysis. Springer-Verlag New York, 2016

