# Supplementary material for: Capture, analyse, visualise: An exemplar of performance analysis in practice in field hockey
Source: PLoS One. 2022 May 5;17(5):e0268171. doi: 10.1371/journal.pone.0268171 (PMC9070925; doi:10.1371/journal.pone.0268171)
Supplement: S1 File — (DOCX) [file pone.0268171.s001.docx]

**Hyperlinks to R code and Output:**

Raw Data and Data analysis R code -<https://github.com/felicitylord/Hockey-Analysis>

Game Styles Instructions and Output -<https://rpubs.com/felicitylord/game-styles>

Ball Movements Instructions and Output -<https://rpubs.com/felicitylord/ball-movements>

In-game events Instructions and Output -<https://rpubs.com/felicitylord/in-game-events>

Shiny App R code -<https://github.com/felicitylord/Hockey-Analysis>

Shiny App -<https://felicity4.shinyapps.io/Hockey101/>
